# Expression of *LOXL3*, *NES*, and *SNAI1* in Melanoma Genesis and Progression

**DOI:** 10.3390/cells13171450

**Published:** 2024-08-29

**Authors:** Zdenka Šitum Čeprnja, Nela Kelam, Marin Ogorevc, Anita Racetin, Martina Vukoja, Toni Čeprnja, Natalija Filipović, Mirna Saraga-Babić, Katarina Vukojević

**Affiliations:** 1 Department of Dermatovenerology, University Hospital of Split, 21000 Split, Croatia; zdenka.situm.ceprnja@mefst.hr; 2 Department of Anatomy, Histology and Embryology, University of Split School of Medicine, 21000 Split, Croatia; nela.kelam@mefst.hr (N.K.); marin.ogorevc@mefst.hr (M.O.); amuic@mefst.hr (A.R.); natalija.filipovic@mefst.hr (N.F.); katarina.vukojevic@mefst.hr (K.V.); 3 Laboratory of Morphology, Department of Histology and Embryology, School of Medicine, University of Mostar, 88 000 Mostar, Bosnia and Herzegovina; martina.vukoja@mef.sum.ba; 4 Department of Pathology, Forensic Medicine and Cytology, University Hospital of Split, 21000 Split, Croatia; tceprnja@mefst.hr; 5 Center for Translational Research in Biomedicine, University of Split School of Medicine, 21000 Split, Croatia

**Keywords:** dysplastic nevus, melanoma, LOXL3, NES, SNAI1, BRAF

## Abstract

Melanoma is the most severe type of skin cancer and among the most malignant neoplasms in humans. With the growing incidence of melanoma, increased numbers of therapeutic options, and the potential to target specific proteins, understanding the basic mechanisms underlying the disease’s progression and resistance to treatment has never been more important. LOXL3, SNAI1, and NES are key factors in melanoma genesis, regulating tumor growth, metastasis, and cellular differentiation. In our study, we explored the potential role of LOXL3, SNAI1, and NES in melanoma progression and metastasis among patients with dysplastic nevi, melanoma in situ, and *BRAF*+ and *BRAF*− metastatic melanoma, using immunofluorescence and qPCR analysis. Our results reveal a significant increase in LOXL3 expression and the highest NES expression in *BRAF*+ melanoma compared to *BRAF*−, dysplastic nevi, and melanoma in situ. As for SNAI1, the highest expression was observed in the metastatic melanoma group, without significant differences among groups. We found co-expression of LOXL3 and SNAI1 in the perinuclear area of all investigated subgroups and NES and SNAI1 co-expression in melanoma cells. These findings suggest a codependence or collaboration between these markers in melanoma EMT, suggesting new potential therapeutic interventions to block the EMT cascade that could significantly affect survival in many melanoma patients.

## 1. Introduction

A malignant tumor derived from melanocytes, melanoma mostly occurs in the skin but can also originate from mucosal surfaces, the ciliary body, the uvea, and conjunctiva of the eye, as well as the leptomeninges. It is the fifth most common malignancy in men and sixth most common in women. Over the past 40 years, melanoma incidence has increased globally at a rate of about 3–7% annually. Melanoma is the most serious type of skin cancer, accounting for almost 90% of deaths related to cutaneous malignancies because of its potential for metastasis. Survival rates depend on the stage of the disease at the time of diagnosis, so early recognition and treatment are crucial to improve the outcome and survival. The 5-year survival rate for localized primary melanomas is 99%. Patients with regional disease have a 5-year survival rate of around 68%, while those with distant metastatic disease around 30% [1,2,3]. Thus, melanoma is a life-threatening disease when diagnosed at an advanced stage or when metastasis occurs after surgical treatment of the primary tumor [4]. The initial oncogenic events in melanoma formation impact genes involved in the mitogen-activated protein kinase (MAPK) and phosphoinositide 3-kinase (PI3K) signaling pathways. These genes include *BRAF*, *NRAS*, and *KIT*. The MAPK signaling pathway is essential in the pathogenesis of various types of cancer as it regulates key cellular functions, including proliferation, growth, apoptosis, transformation, and migration [5,6,7,8]. The protein RAS binds to the RAS-binding domain of RAF, leading to dimerization and activation of RAF, which subsequently phosphorylates MEK and leads to its activation and the activation of MAPK [9]. The presence of an oncogenic *BRAF* mutation results in continuous *BRAF* activity and the activation of the MEK and MAPK pathways, independent of the inclusion of RAS/RAF [10]. The V600E mutations of *BRAF*, namely the substitution of valine with glutamic acid at codon 600, were detected in over 90% of acquired melanocytic nevi and over 50% of cutaneous melanomas [11,12,13,14]. *BRAF* or NRAS activity leads to cellular proliferation and nevus formation with limited growth. However, benign nevi can progress to melanoma, and studies have shown a linear progression pattern from precursor lesions like benign and dysplastic nevi to melanoma in situ and invasive melanoma [5,15]. Specific molecular events characterize this transition. Among the various factors implicated in melanoma genesis, the roles of lysyl oxidase-like 3 (LOXL3), Snai1 family transcriptional repressor 1 (SNAI1), and nestin (NES) have garnered significant attention due to their involvement in key pathways that regulate tumor growth, metastasis, and cellular differentiation [16,17]. The LOXL3 enzyme belongs to the lysyl oxidase family, which consists of five associated members: the prototype LOX and four LOX-like enzymes, LOXL1–4. These enzymes control the balance of the extracellular matrix and play a role in the development of tumors through intracellular and extracellular actions [18]. They function as extracellular enzymes, facilitating cross-linking and stabilizing collagen and elastin fibers. Nevertheless, research has also demonstrated their participation in gene transcription, epithelial–mesenchymal transition (EMT), development, differentiation, and angiogenesis in cancer [17,19]. Prior research has demonstrated that LOXL3 is crucial for the survival of human melanoma cells and the preservation of genomic stability. Animal models with LOXL3 deletion exhibited prolonged onset of melanoma, reduced tumor development, and decreased spread of metastases. It was proposed that LOXL3 modulates phenotypic switching through SNAI1 [20]. NES is a type VI intermediate filament protein first identified as a marker for neural stem cells. The expression of this gene was detected in embryonic cells, and its association with malignancies has been documented in melanoma as well as other tumors, including ovarian cancer, breast cancer, prostate cancer, pancreatic cancer, and osteosarcoma [21,22,23,24]. NES expression in melanoma has been associated with cell migration, invasion, disease progression, and metastasis. It is expressed more strongly in melanomas compared to benign nevi, and even more in advanced melanoma stages [25,26]. SNAI1 is a member of the Snai1 superfamily of zinc finger transcription factors that govern the EMT of various cancers. An increase in SNAI1 expression was observed to correlate with tumor progression and recurrence. SNAI1 is overexpressed in melanoma, hepatocellular carcinoma, head and neck squamous cell carcinoma, and endometrial cancers [27,28,29,30]. In animal models, it has been shown that Snai1 depletion blocks melanoma growth by interrupting cell proliferation and enhancing apoptosis [31].

Considering that the co-expression of LOXL3, SNAI1, and NES in melanoma has not been described in the existing literature, our study aimed to determine whether LOXL3, SNAI1, and NES are potential risk factors and whether their expression can be used to predict melanoma progression and metastasis in patients with dysplastic nevi, melanoma in situ, and *BRAF*+ and *BRAF*− metastatic melanoma. Our results can potentially improve comprehension of the association between the investigated factors and melanoma progression, which could lead to the development of potential new therapeutic targets for melanoma treatment.

## 2. Materials and Methods

### 2.1. Tissue Procurement and Processing

A total of 45 paraffin blocks of skin biopsy samples with a pathological diagnosis of dysplastic nevus, melanoma in situ, and *BRAF*+ and *BRAF−* melanoma were collected from the Department of Pathology, Forensic Medicine and Cytology at the University Hospital Center Split from 2017 to 2020 (Table 1). Ethical approval was given by the Ethics Committee of University Hospital Center Split (class: 520-03/24-01/95, approval number: 2181-147/01-06/LJ.Z.24-02), following the Helsinki Declaration [32]. The pathology report was used to derive clinical data from the time of the biopsy. Inclusion criteria demanded sufficient paraffin block material for immunohistochemistry (IHC) and complete clinical data. Incomplete laboratory results or an insufficient amount of tissue for IHC constituted the exclusion criteria.

The skin biopsy samples were collected and fixed with 4% paraformaldehyde (PFA) in 0.1 M phosphate buffer saline (PBS) overnight to enable standard histological examinations, including hematoxylin–eosin and immunofluorescence labeling. After dehydration of the tissue in graded ethanol solutions and clearing in xylol, it was embedded in paraffin blocks, serially cut to a thickness of 5 µm using a microtome, and mounted on glass slides. Every tenth section was stained by hematoxylin–eosin (H&E). Proper tissue preservation and pathological findings in the skin biopsies were examined by light microscopy.

### 2.2. Immunofluorescence

Following deparaffinization using xylol and the gradual rehydration in water–ethanol solutions, the histological slides were subjected to antigen retrieval. This involved heating the slides in a water steamer at 95 °C for 30 min in a 0.01 M citrate buffer (pH 6.0), followed by cooling to room temperature. After washing the slides in a 0.1 M PBS solution, a protein-blocking solution (ab64226, Abcam, Cambridge, UK) was applied for 30 min at room temperature to prevent non-specific staining. Table 2 provides comprehensive information involving the application and overnight incubation of primary antibodies in a humidity chamber. This was followed by rinsing in PBS and a subsequent one-hour incubation of secondary antibodies (Table 2). The nuclei were detected with 4′,6-diamidino-2-phenylindole (DAPI) after the slides were washed with PBS. Following the last rinse with distilled water, the mounting media (Immumount, Shandon, Pittsburgh, PA, USA) was applied, and the histological slides were covered with a cover slip.

Prior to the preadsorption experiment, each primary antibody was diluted to the predetermined concentration using a blocking solution. Tissue slices were treated with a solution containing a suitable peptide antigen. No antibody staining was seen. When the primary antibodies were excluded from the experimental technique, there were no signs of non-specific binding of the secondary antibody or any false-positive results.

### 2.3. Data Acquisition

H&E slides were analyzed using a light microscope (BX40, Olympus, Tokyo, Japan). Images of the skin biopsy samples were recorded by an epifluorescence microscope (BX51, Olympus, Tokyo, Japan) equipped with a Nikon DS-Ri2 camera (Nikon Corporation, Tokyo, Japan) with NIS-Elements F software (version 5.22.00). LOXL3, SNAI1, and NES were analyzed in ten non-overlapping representative fields at ×1000 total magnification with the use of immersion oil (Carl Roth, Karlsruhe, Germany) with a constant exposure duration. Green staining was interpreted as positive LOXL3 and NES immunoexpression, and red staining as positive SNAI1 immunoexpression.

### 2.4. Semi-Quantification

The intensity of staining of LOXL3, SNAI1, and NES markers in dysplastic nevus, melanoma in situ, *BRAF+* and *BRAF−* melanomas were semi-quantitatively evaluated into four groups according to the staining reactivity: no reactivity = −, mild reactivity = +, moderate reactivity = ++, and strong reactivity = +++.

### 2.5. Image Analysis of Area Percentage

The microphotographs were processed and analyzed using ImageJ software Version 1.54 (National Institutes of Health, Bethesda, MD, USA) for quantitative cell assessment of immunoreactivity as described previously [33,34,35]. The fluorescence leak was decreased by subtracting the red countersignal from the green fluorescence and applying a median filter with a 2.0-pixel radius. Each image was then altered using the threshold method (triangle thresholding algorithm), and the “analyze particles” option was used to measure the fluorescence percentage area.

### 2.6. Statistical Analysis of Area Percentage

GraphPad Prism 9.0.0 software was used for statistical analyses (GraphPad Software, San Diego, CA, USA). The Shapiro–Wilk test was used to check whether the data distribution was normal. Each dataset regarding area percentage analysis was described with *p* at the probability level of *p* < 0.05, regarded as statistically significant. Datasets were analyzed using an ordinary one-way ANOVA followed by Tukey’s multiple comparisons test. The percentage of positive cells was expressed as the mean ± standard deviation (SD).

All graphs were generated using GraphPad Prism 9.0.0. Plates were created using Adobe Photoshop version 21.0.2 (Adobe, San Francisco, CA, USA). Background subtraction and contrast were applied to microphotographs for presentation purposes.

### 2.7. RNA Isolation and Reverse Transcription

Total RNA was extracted from 24 human formalin-fixed, paraffin-embedded melanoma samples, including dysplastic nevus (n = 6), melanoma in situ (n = 6), *BRAF+* (n = 6), and *BRAF−* (n = 6). Multiple 6 µm thick tissue slices were placed in RNAse-free tubes and processed with High Pure RNA Paraffin (Cat. No. 03270289001; Roche, Basel, Switzerland) following the manufacturer’s instructions. The protocol began with deparaffinizing the paraffin-embedded tissue, washing it in absolute ethanol, and centrifuging at maximum speed for 2 min. Proteinase K and Tissue Lysis Buffer were then added to the dried pellet for digestion and overnight incubation. The following day, the binding buffer and ethanol were applied to the lysate, and the solution was applied to a spin column. The bound RNA was washed from the column, and DNase working solution and incubation buffer were added to the eluate and mixed. The total RNA in each sample was quantified using the Qubit™ 4 Fluorometer (Thermo Fisher Scientific Inc., Waltham, MA, USA). The samples were diluted to match the lowest measured concentration (1.34 ng/µL). Then, 1.34 nanograms of total RNA was reverse transcribed into complementary DNA (cDNA) using a High Capacity Reverse Transcriptase Kit (Applied Biosystems, Foster City, CA, USA) with random primers, as per the manufacturer’s instructions. The cDNA, with a final volume of 20 µL, was stored at −80 °C for subsequent quantification of genes of interest.

### 2.8. qPCR

qPCR analysis was conducted on a Real-Time PCR instrument (Applied BiosystemsFast 7500, Waltham, MA, USA) using Taqman^®^ Fast Advanced Universal Master Mix II (Applied Biosystems, Waltham, MA, USA) comprising AmpEraseuracil-N-glycosylase and the passive reference dye ROX. ProbesTaqman^®^ gene expression assays for human *LOXL3*, *SNAI1*, and *NES* were provided by Applied Biosystems (Hs01046945_m1, Hs00195591_m1, Hs01895061_u1, and Hs00707120_s). Glyceraldehyde-3-phosphate dehydrogenase (GAPDH) was used as the housekeeping gene (assay ID Hs99999905_m1). Taqman real-time PCR was performed with a 2 µL cDNA template, 1 µL Taqman^®^ (Applied Biosystems, Waltham, MA, USA) gene expression assay, and 10 µL Taqman^®^ (Applied Biosystems, Waltham, MA, USA) universal master mix, bringing the total volume to 20 µL. The PCR protocol involved an initial 2 min incubation at 50 °C for uracil-N-glycosylase activation, followed by 2 min at 95 °C for polymerase activation, and then 40 cycles of amplification (3 s at 95 °C and 30 s at 60 °C). Duplicate PCRs were conducted for each gene per cDNA sample. A negative control with nuclease-free water instead of a cDNA template was included in each experiment. The 2−∆∆CT method was employed for relative quantification. The plate was subsequently analyzed using the Applied Biosystems™ 7500 RT-PCR system (Thermo Fisher Scientific, Waltham, MA, USA).

### 2.9. Statistical Analysis of RT-qPCR

Statistical analysis was conducted using ordinary one-way ANOVA followed by Tukey’s multiple comparisons test, utilizing Prism 9.0.0 for Windows (GraphPad Software, San Diego, CA, USA). Data were expressed as mean ± SD, with *p* < 0.05 considered statistically significant. To implement the 2^−ΔΔCt^ method, the average ΔCt values from dysplastic nevus samples served as a calibrator for calculating the relative fold gene expression of all samples relative to dysplastic nevus.

### 2.10. Transcriptomics

Differential expression of the *LOXL3*, *NES*, and *SNAI1* genes between melanoma and normal skin samples, as well as survival analysis based on their expression status, was performed using the standard processing pipeline of the publically available database Gene Expression Profiling Interactive Analysis 2 (GEPIA2, http://gepia2.cancer-pku.cn/, accessed on 15 June 2024) [36]. The data sources for the analyses performed in GEPIA2 were the TCGA Skin Cutaneous Melanoma (SKCM) and GTEx Skin datasets. The Subtype Filter function was used to separately analyze TCGA SKCM samples with (N = 147) and without (N = 165) confirmed *BRAF* mutations. Differential expression analysis was performed using one-way ANOVA with cutoff values |log2FC| ≥ 1 and *p* < 0.01. Overall survival analysis based on the expression of the analyzed genes was performed between the lowest and highest 50% for each gene using the Log-rank test with significance set at *p* < 0.05. The box-plots for differential expression and Kaplan–Meier curves of overall survival were constructed in GEPIA2.

## 3. Results

Morphological differences between dysplastic nevi, melanoma in situ, and *BRAF−* and *BRAF*+ melanoma were observed in H&E slides. Using immunofluorescence, LOXL3, NES, and SNAI1 expression was evaluated with different expression patterns and intensity. Quantitative cell evaluation of LOXL3, NES, and SNAI1 immunoreactivity in dysplastic nevi, melanoma in situ, and *BRAF−* and *BRAF+* melanoma was performed by determining the section percentage area of the epidermal region. The results were presented as the percentage of the area showing a positive signal.vRT-qPCR analysis of FFPE skin biopsies was performed on the same specimens to determine the fold gene expression score of observed mRNAs between dysplastic nevi, melanoma in situ, and *BRAF−* and *BRAF+* melanoma. The *LOXL3*, *NES,* and *SNAI1* in high and low expressions in the TCGA SKCM study were analyzed for the survival rate and the average survival time.

### 3.1. H&E Staining of Dysplastic Nevus, Melanoma In Situ, and BRAF− and BRAF+ Melanoma

Histological examination of dysplastic nevi revealed architectural disorder and cytologic atypia. Architectural disorder included the presence of junctional shoulders adjacent to a dermal component, bridging of nests between adjacent elongated rete ridges, suprabasal scatter of melanocytes confined to the lower epidermal levels, concentric and lamellar fibroplasia around elongated rete ridges, and a patchy lymphocytic infiltrate. Cytologic atypia was characterized by enlargement of nuclei with varying degrees of irregularity, chromatin clumping and hyperchromatism, and variably prominent nucleoli.

**Figure 1:**
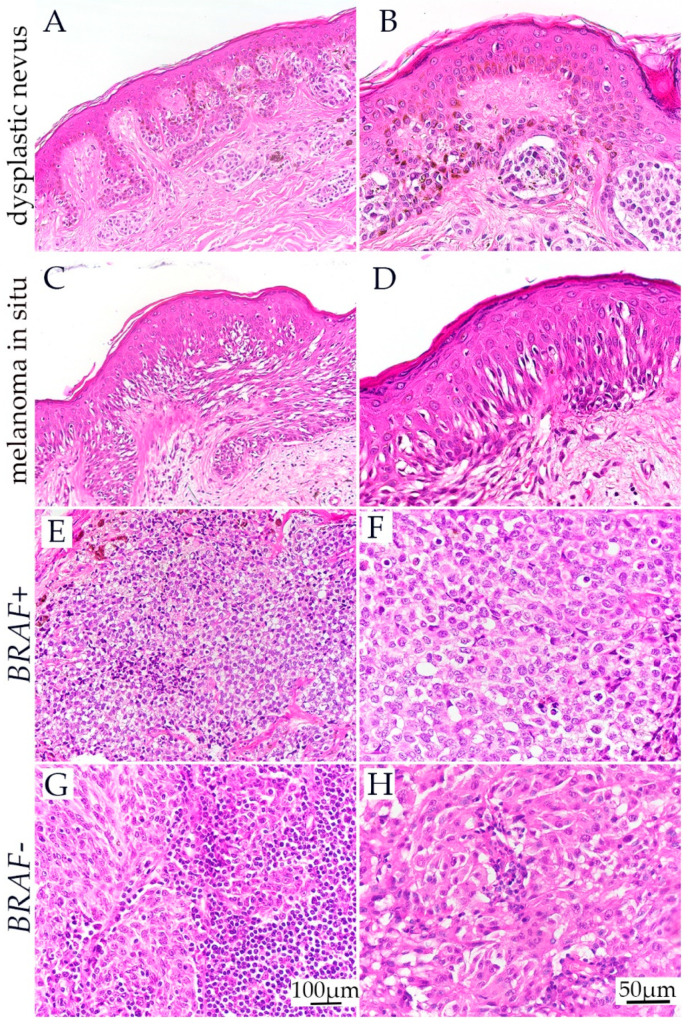
Hematoxylin and eosin staining of different melanocytic lesions included in the study. Dysplastic nevus characterised by an architectural disorder (nest bridging) and moderate cytological atypia (**A**,**B**). The confluent proliferation of melanocytes with severe atypia along the base of the epidermis with intraepidermal proliferation (pagetoid scatter), but with no apparent invasive component in melanoma in situ (**C**,**D**); Invasive components of *BRAF*+ and *BRAF−* invasive melanomas consisting of cohesive aggregates of neoplastic melanocytes (**E**–**H**).

Similar atypical characteristics were also seen in melanoma in situ and invasive melanomas. However, these were accompanied by either high-level and/or extensive pagetoid scatter or extensive continuous basal proliferation of atypical melanocytes, more severe and more uniform cytologic atypia, mitotic activity, and in cases of invasive melanoma, failure of maturation of the dermal component (Figure 1).

### 3.2. LOXL3 Expression

**Figure 2:**
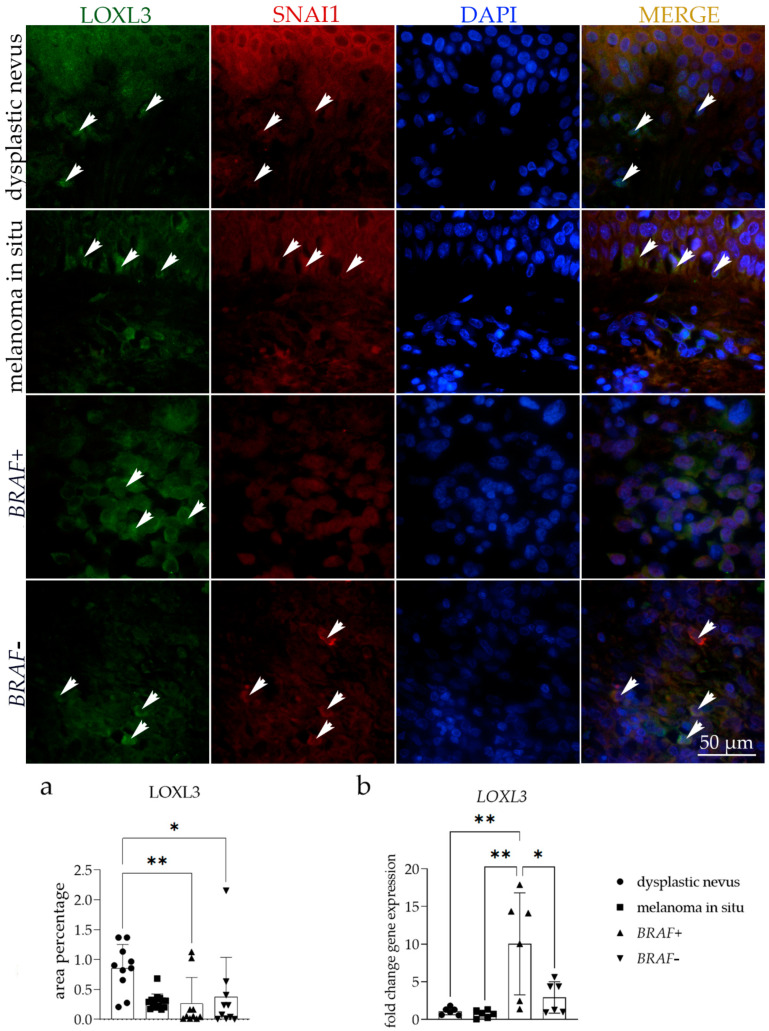
Immunoexpression of lysyl oxidase homolog 3 (LOXL3), zinc finger protein SNAI1 (SNAI1), 4′,6-diamidino-2-phenylindole (DAPI) nuclear staining image, and merged LOXL3, SNAI1, and DAPI staining in dysplastic nevus, melanoma in situ, and *BRAF−* and *BRAF*+ melanoma. The arrows show the expression pattern of LOXL3 and SNAI1 in the skin tissue. The arrows on the merged microphotographs indicate the area where co-expression was observed. Images were captured at a magnification of ×1000, with a scale bar of 50 µm applicable to all images. The LOXL3 area percentages in the tissue of dysplastic nevus, melanoma in situ, and *BRAF−* and *BRAF+* melanoma (**a**). Data are presented as the mean ± SD (vertical line) and analyzed by an ordinary one-way ANOVA followed by Tukey’s multiple comparisons test. At each time point, ten representative pictures were assessed. The *LOXL3* mRNA fold change gene expression comparison between dysplastic nevus, melanoma in situ, and *BRAF−* and *BRAF*+ melanoma (**b**). Ordinary one-way ANOVA was followed by Tukey’s multiple comparison test. Data are shown as mean ± SD (vertical line); significant differences are marked by * *p* < 0.05, ** *p* < 0.01.

The immunohistochemical staining pattern of LOXL3 ranged from negative to punctate nuclear and cytoplasmic staining in all tissue samples examined (Table 3). The LOXL3 staining signal was widely distributed in all layers of the epidermis, in the cells of the dysplastic nevus nests, and in the tumor cells in the basal layer of the epidermis of melanoma in situ samples (Figure 2). Compared to other observed groups, the strongest immunoreactivity was noticed in the epithelium of *BRAF+* and *BRAF−* melanoma samples (Table 3).

Co-expression of LOXL3 and SNAI1 was noticed sporadically in the perinuclear area of all observed phenotypes.

The area percentage of LOXL3 was significantly higher in dysplastic nevus compared to *BRAF+* (*p* = 0.0061) and *BRAF−* melanoma (*p* = 0.0270) (Figure 2a). The RT-qPCR analysis demonstrated a significantly higher *LOXL3* fold change gene expression in the *BRAF*+ melanoma than in the *BRAF−* melanoma (*p* = 0.0214), dysplastic nevus (*p* = 0.0035), and melanoma in situ (*p* = 0.0024) (Figure 2b).

### 3.3. NES Expression

**Figure 3:**
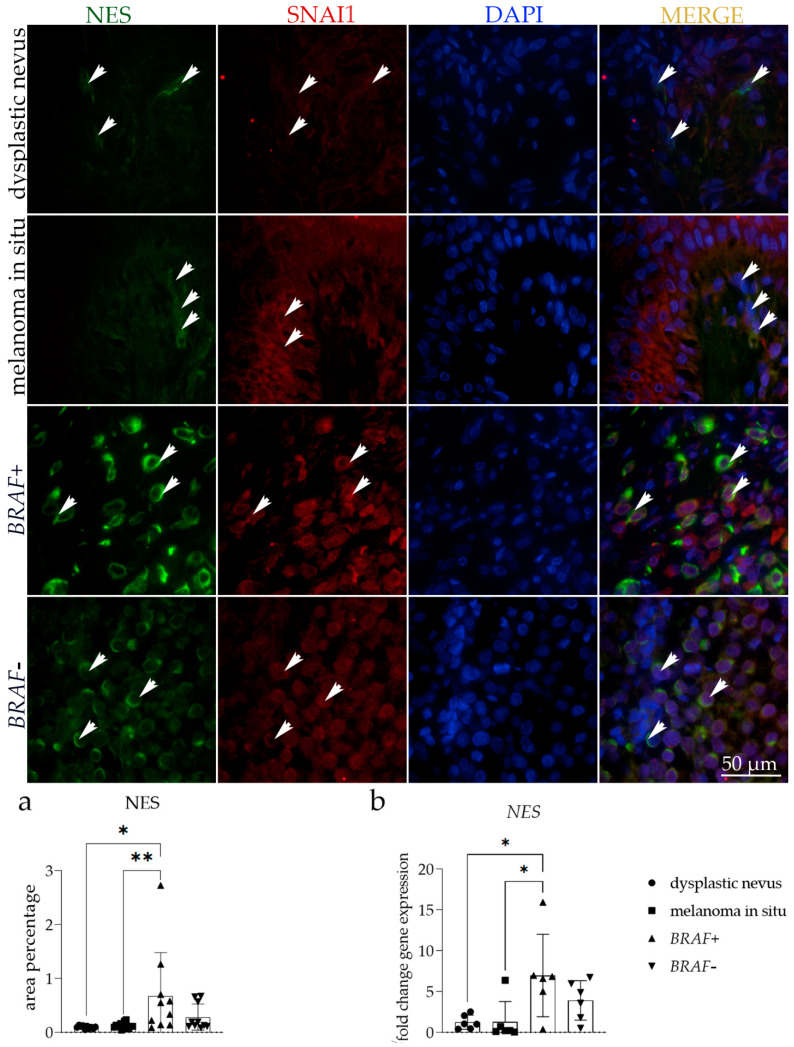
Immunoexpression of nestin (NES), zinc finger protein SNAI1 (SNAI1), 4′,6-diamidino-2-phenylindole (DAPI) nuclear staining image, and merged NES, SNAI1, and DAPI staining in dysplastic nevus, melanoma in situ, and *BRAF−* and *BRAF*+ melanoma. The arrows show the NES and SNAI1 expression patterns in the skin tissue. The arrows on the merged microphotographs indicate the area where co-expression was observed. Images were captured at a magnification of ×1000, with a scale bar of 50 µm applicable to all images. The NES area percentages in the tissue of dysplastic nevus, melanoma in situ, and *BRAF−* and *BRAF*+ melanoma (**a**). Data are presented as the mean ± SD (vertical line) and analyzed by an ordinary one-way ANOVA followed by Tukey’s multiple comparisons test. At each time point, ten representative pictures were assessed. The *NES* mRNA fold change gene expression comparison between dysplastic nevus, melanoma in situ, and *BRAF−* and *BRAF*+ melanoma (**b**). Ordinary one-way ANOVA was followed by Tukey’s multiple comparison test. Data are shown as mean ± SD (vertical line); significant differences are marked by * *p* < 0.05, ** *p* < 0.01.

Nestin staining was noticed as mild diffuse staining appearing sporadically in tumor cells of irregular nests of dysplastic nevus and melanoma in situ. In *BRAF*+ melanoma, however, strong diffuse cytoplasmic staining was noticed (Figure 3). Compared to other observed groups, the strongest immunoreactivity was seen in the epithelium of *BRAF+* melanoma and moderate in the *BRAF−* melanoma samples (Table 3).

Nestin and SNAI1 were co-expressed in the melanoma cells (Figure 3).

The area percentage and the RT-qPCR analysis demonstrated similar results for all observed groups. The *BRAF+* melanoma showed a substantially higher NES area percentage compared to dysplastic nevus (*p* = 0.0201) and melanoma in situ (*p* = 0.0160) (Figure 3a).

When we compared the *NES* mRNA fold change gene expression in all tissues examined, we detected a significantly higher expression score in *BRAF*+ melanoma than in dysplastic nevus (*p* = 0.0357) and melanoma in situ (*p* = 0.0367) (Figure 3b).

### 3.4. SNAI1 Expression

SNAI1 staining patterns demonstrated punctate nuclear and cytoplasmic staining in all tissue samples examined (Figure 2 and Figure 3). Semi-quantitative analysis demonstrated the moderate immunoreactivity in the epithelium of *BRAF+* and *BRAF−* melanoma samples, compared to other observed sample groups (Table 3).

**Figure 4:**
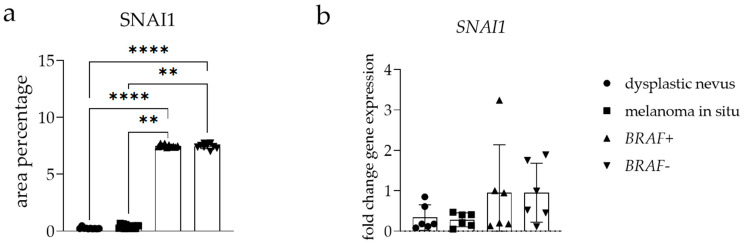
The zinc finger protein SNAI1 (SNAI1) area percentages in the tissue of dysplastic nevus, melanoma in situ, and *BRAF−* and *BRAF*+ melanoma (**a**). Data are presented as the mean ± SD (vertical line) and analyzed by an ordinary one-way ANOVA followed by Tukey’s multiple comparison test. At each time point, ten representative pictures were assessed. The *SNAI1* mRNA fold change gene expression comparison between dysplastic nevus, melanoma in situ, and *BRAF−* and *BRAF*+ melanoma (**b**). Ordinary one-way ANOVA was followed by Tukey’s multiple comparison test. Data are shown as mean ± SD (vertical line); significant differences are marked by ** *p* < 0.01, **** *p* < 0.0001.

The area percentage of SNAI1-positive cells showed a significantly higher area percentage score in *BRAF*+ and *BRAF−* melanoma samples than in dysplastic nevus (*p* < 0.01) and melanoma in situ samples (*p* < 0.0001) (Figure 4a).

The RT-qPCR analysis demonstrated the expression of *SNAI1* mRNA in all tissues tested, with no significant difference in the fold change gene expression comparing the tissues examined. The highest *SNAI1* mRNA expression was observed in *BRAF*+ and *BRAF−* melanoma (Figure 4b).

### 3.5. Differential Expression

**Figure 5:**
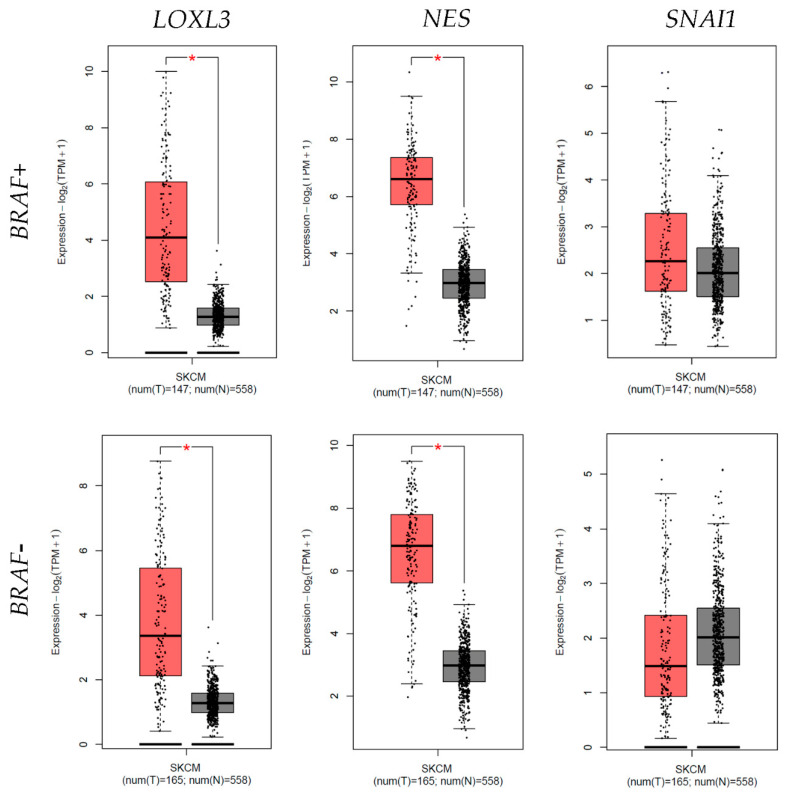
Graphic representation of the differential expression of lysyl oxidase homolog 3 (*LOXL3*), nestin (*NES*), and zinc finger protein SNAI1 (*SNAI1*) mRNA in *BRAF*+ and *BRAF−* melanoma (red colored bars) compared to normal skin samples (grey colored bars). Statistically significant differences were found in LOXL3 and NES mRNA differential expression in both *BRAF*+ and *BRAF−* melanoma. Data are used from the TCGA Melanoma (SKCM) study. Significant differences are marked by * *p* < 0.05.

The differential expression analyses perfomed using GEPIA2 revealed a significantly higher mRNA expression level for *LOXL3* and *NES* in cutaneous melanomas, both with and without confirmed *BRAF* mutations, compared to normal skin samples. There were no significant differences in *SNAI1* mRNA expression between melanoma samples and normal skin, regardless of the *BRAF* status (Figure 5).

### 3.6. Survival Analysis

**Figure 6:**
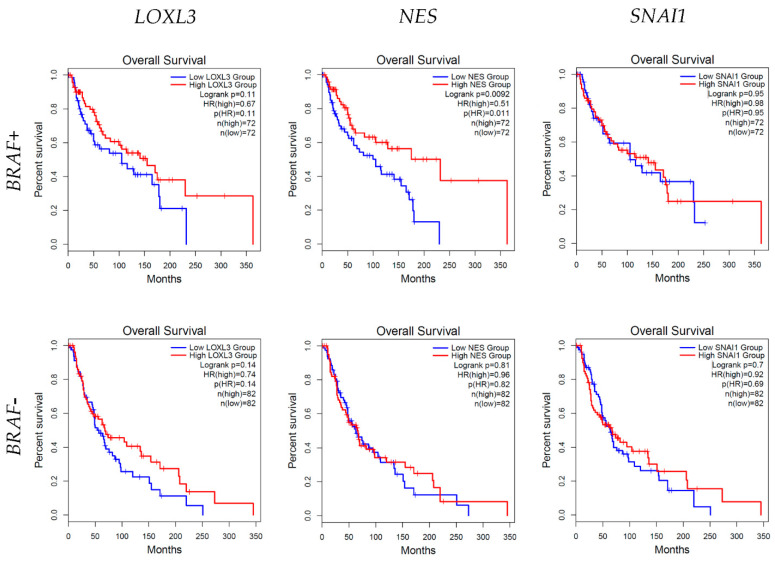
Graphical representation of survival analysis of lysyl oxidase homolog 3 (*LOXL3)*, nestin (*NES)* and zinc finger protein (*SNAI1)* in high (red line) and low (blue line) mRNA expression in *BRAF*+ and *BRAF−* melanoma was expressed as the average survival time in months. A statistically significant difference (*p* = 0.0092) in survival times was found between the high- and low-*NES*-expression groups. The Kaplan–Meier method and Log-rank test for survival length were used. Data are utilized from the TCGA Melanoma (SKCM) study.

The survival rates in relation to the high and low mRNA expressions of *LOXL3*, *NES*, and *SNAI1* in both *BRAF*-positive (*BRAF*+) and *BRAF*-negative (BRAF−) melanoma were analyzed (Figure 6).

In *BRAF*+ melanoma, there was no significant difference in the survival times between the high- and low-expression groups of *LOXL3* and *SNAI1*. However, a statistically significant difference (*p* = 0.0092) in survival times was found between the high- and low-*NES*-expression groups (Figure 6).

In contrast, for *BRAF−* melanoma, no statistically significant differences were observed in survival times between the high- and low-expression groups of *LOXL3*, *NES*, and *SNAI1* (*p* = 0.14, *p* = 0.81, and *p* = 0.07, respectively) (Figure 6).

## 4. Discussion

With the growing incidence of melanoma, an increased number of therapeutic options, and the potential to target specific proteins, understanding tumorigenesis has never been more important. Upon reviewing the literature, it becomes apparent that certain markers, such as LOXL3, SNAI1, and NES, are associated with melanoma development. However, their specific roles, as well as the expression and co-expression of these markers in different melanoma groups and dysplastic nevi as melanoma precursors, have not been thoroughly examined.

Our study, using two experimental methods, immunofluorescence and qPCR analysis, reports on the aberrant *LOXL3*, *SNAI1*, and *NES* expression and their co-expression in dysplastic nevi, melanoma in situ, and *BRAF*+ and *BRAF*– melanoma. The same techniques in analyzing *Loxl3* influence on melanoma progression and dissemination were used in a recent study by Vázquez-Naharro on animal models, and it showed that *Loxl3* knockout and activation of *Braf* with concomitant inactivation of *Pten* reduced melanoma growth, while increasing latency and overall mice survival. Furthermore, in a melanoma cell lines study, *Loxl3*-silenced cells showed a marked decrease in *Snai1* compared to the control. This finding was also confirmed on the protein level, indicating that Loxl3 might regulate Snai1 expression and activity [20], which is consistent with earlier studies showing that overexpression of Lox2 induces EMT via Snai1, implicating a similar role for LOXL3 [37]. NES was depicted as a reliable factor in prognosis melanoma metastasis, and a study demonstrated that reducing nestin expression decreases cell growth, migration, and invasion in human melanoma cells [21,38].

Our study demonstrated a substantial increase in the expression of the *LOXL3* in *BRAF−*positive melanoma compared to *BRAF−*negative melanoma, dysplastic nevi, and melanoma in situ. This finding suggests that *LOXL3* has a significant role in melanoma genesis from precursor lesions and in situ tumors to metastatic melanoma and corroborates findings from previous studies [20,39]. The association between the overexpression of LOXL3 and the transformation of immortalized human melanocytes carrying the *BRAF* V600E mutation into malignant cells was originally described in an in vitro experiment on melanoma cell lines [39]. Reports from a study on animal models also refer to the interaction of *Loxl3* and oncogene *Braf*, one of the most common mutations found in human melanoma, to facilitate tumor development and progression [20]. The study of Zhang et al. discovered a significant association between the expression of LOXL3 and the growth and depth of invasion of primary melanoma. Furthermore, they observed that patients with higher LOXL3 expression had a poorer prognosis [40]. This finding does not align with our hypothesis, since we found higher LOXL3 expression in dysplastic nevi compared to all investigated melanoma subtypes. One of the possible explanations is that this could be due to the fact that our study used dysplastic nevi, which are considered to be precursors of melanoma, instead of healthy skin as the reference point [5,15].

The highest NES expression in our study was noticed in *BRAF*+ melanoma, compared to dysplastic nevi and melanoma in situ, while there were no significant differences between the *BRAF−* group and others. Concerning the mRNA expression, *NES* was expressed significantly higher in *BRAF*+ melanoma than in dysplastic nevus and melanoma in situ. These results align with previous studies’ findings and suggest that this marker plays a significant role in advancing malignancies, with NES expression increasing during each step of proposed tumor development [21,25,26].

Furthermore, metastases tend to have higher levels of NES than primary tumors [21,26]. To the best of our knowledge, our study is the first to investigate the expression of these markers in dysplastic nevi. In the future, it would be interesting to see if there is a difference in nestin expression among benign nevi and dysplastic ones.

We also investigated SNAI1 expression among dysplastic nevi and melanoma subgroups and revealed higher SNAI1 expression in *BRAF*+ and *BRAF*– melanoma samples than in dysplastic nevi and melanoma in situ. This finding is in line with previous research investigating the influence of *SNAI1* overexpression on human melanoma cell lines in hypoxic conditions, which revealed that elevated SNAI1 expression leads to the acquisition of cancer stem cell-like characteristics and an enhanced ability to metastasize [41]. Additionally, we have demonstrated the expression of *SNAI1* mRNA in all tissues tested. The expression trend was similar and followed the immunohistochemistry results: the highest *SNAI1* mRNA expression was observed in *BRAF*+ and *BRAF*– melanomas, supporting the role of SNAI1 as a potent epithelial repressor that cancer cells activate to detach from neighboring cells [20,42]. However, we found no significant difference when comparing investigated groups. It is plausible that the mRNA detected by PCR is not efficiently translated into protein or that the resulting protein undergoes post-translational modifications, degradation, or changes in localization that render it less detectable by IHC. Conversely, proteins identified through IHC may exhibit longer half-lives, resulting in sustained detectability even when corresponding mRNA levels are reduced [43]. Additionally, the limited sample size may contribute to variability in the results, potentially amplifying discrepancies between the two methods.

Furthermore, we compared the RT-qPCR results of the observed genes from our samples with the mRNA expression data found in the TCGA melanoma database. When analyzing differential expression using GEPIA2, we have found a significantly higher expression for *LOXL3* and *NES* in cutaneous metastatic melanoma, both in *BRAF*+ and *BRAF*– tumors, compared to normal skin samples. Although statistical validation of our findings would require analyses of larger sample cohorts, the data from our samples suggest significantly higher *LOXL3* fold change gene expression in *BRAF*+ melanoma than in *BRAF*– melanoma, dysplastic nevi, and melanoma in situ. As for *NES,* we found higher expression in *BRAF*+ melanoma than in *BRAF−* melanoma and significantly higher expression than in dysplastic nevus and melanoma in situ, emphasizing the significance of *NES* in melanoma progression. However, our analysis of differential expression on the data from the TCGA melanoma study showed no differences in *SNAI1* expression between melanoma samples and normal skin, regardless of *BRAF* status. We have also not observed any significant differences in *SNAI1* expression between the investigated groups of our samples. Results showed the highest *SNAI1* mRNA expression in metastatic melanoma, regardless of BRAF status. The fact that the mRNA expression analyses of all three genes from our limited number of samples correspond to those from the much larger TCGA melanoma database indicates that our samples are relatively representative.

The major point of our interest was to explore the co-expression of LOXL3, SNAI1, and NES in melanoma development and their supposed role in EMT. During our search of available literature, no previous studies reported on or investigated the co-expression of these markers, although they were all implicated in the same process of EMT, which is an important milestone of tumor progression. We have found co-expression of LOXL3 and SNAI1 in the perinuclear area of all investigated subgroups, and we have also found NES and SNAI1 co-expression in melanoma cells. These findings, combined with previous knowledge that LOXL3 and SNAI1 facilitate detachment and migration of cells from primary melanoma [20,42], while NES enhances cell motility and structural reorganization [44,45], suggest some level of codependence or collaboration between these markers in melanoma EMT. These results should be investigated further as potential future therapeutic interventions, as we can hypothesize that blocking the cascade that leads to EMT could significantly affect survival in many melanoma patients.

Analysis of the influence of *LOXL3, NES,* and *SNAI1* expression data from the TCGA melanoma database on the survival rates in patients discovered that in the group of *BRAF*+ melanoma patients, there was no significant difference in survival times between the high- and low-expression groups of *LOXL3* and *SNAI1*. However, there was a statistically significant difference in survival rates between high- and low-*NES*-expression groups, favoring high *NES* expression and longer survival. This finding is opposite to the previous studies where NES was significantly increased in melanomas than in melanocytic nevi [46], and it correlated with more advanced stages of the disease, metastatic potential, and poor survival rates [47,48]. However, these studies analyzed the protein expression, not the mRNA expression. As for survival analysis among *BRAF−* melanoma patients, no statistically significant differences were observed in survival times between the high- and low-expression groups of *LOXL3, NES*, and *SNAI1.*

## 5. Conclusions

Our study, although limited because of the small size of our cohort, provides evidence that the expressions of the investigated markers LOXL3, NES, and SNAI1 change with the progression of melanoma. These markers are also co-expressed in melanoma cells. Blocking these specific markers may have an impact on the progression of melanoma and potentially affect the prognosis of many melanoma patients in the future. However, additional studies are needed to further our understanding of these markers’ exact roles and interactions in melanoma.

## Figures and Tables

**Table 1:** Socio-demographic characteristics of patients.

Histology	No Patients	Age	Sex (Male/Female)
Dysplastic nevus	10	36.9 ± 12.2	6/4
Melanoma in situ	15	55.9 ± 10.9	5/5
BRAF+ melanoma	10	60.8 ± 13.1	9/6
BRAF− melanoma	10	65.3 ± 15.9	7/3

**Table 2:** Primary and secondary antibodies used for immunofluorescence.

Antibodies		Catalog Number	Host	Dilution	Source
Primary	Anti-LOXL3	SAB4301652	Rabbit	1:100	Merck KGaA, Darmstadt, Germany
	Anti-SNAI1 antibody	ab53519	Goat	1:500	Abcam, Cambridge, UK
	Anti-nestin antibody (SP103)	ab105389	Rabbit	1:100	Abcam, Cambridge, UK
Secondary	Rhodamine Red™-X (RRX) AffiniPure Anti-Goat IgG (H + L)	705-295-003	Donkey	1:300	Jackson Immuno Research Laboratories, Inc., (Baltimore, PA, USA)
	Alexa Fluor^®^488 AffiniPure Anti- Rabbit lgG (H + L)	711-545-152	Donkey	1:300	Jackson Immuno Research Laboratories, Inc., (Baltimore, PA, USA)

**Table 3:** Immunoreactivity to LOXL3, SNAI1, and NES markers in dysplastic nevus, melanoma in situ, *BRAF+* and *BRAF−* melanoma.

Structure	Antibodies											
	LOXL3				NES				SNAI1			
	Dysplastic Nevus	Melanoma In Situ	BRAF+	BRAF−	Dysplastic Nevus	Melanoma In Situ	BRAF+	BRAF−	Dysplastic Nevus	Melanoma In Situ	BRAF+	BRAF−
epithelium	+	+	++	++	+	+	+++	++	+	+	++	++
lamina propria	+/−	+/−	+	+	+/−	+/−	+	+	+/−	+/−	+	+

+++ strong reactivity; ++ moderate reactivity; + mild reactivity; − no reactivity.

## Data Availability

All data and materials are available upon request. Data regarding the expression of *LOXL3*, *NES*, and *SNAI1* in TCGA Melanoma (SKCM) study can be found at the publicly available websites https://www.genome.gov/Funded-Programs-Projects/Cancer-Genome-Atlas (accessed on 4 June 2024) and https://gtexportal.org/home/ (accessed on 4 June 2024), which contain databases for gene expression in cancers.
